# MiRNA-based model for predicting the TMB level in colon adenocarcinoma based on a LASSO logistic regression method

**DOI:** 10.1097/MD.0000000000026068

**Published:** 2021-05-28

**Authors:** Zhengtian Li, Lingling Jiang, Rong Zhao, Jun Huang, Wenkang Yang, Zhenpei Wen, Bo Zhang, Gang Du

**Affiliations:** a Guangxi Medical University, Nanning; b Department of Anesthesiology, The Second Hospital of Anhui Medical University, Hefei; c Department of Orthopedics Trauma, The Third Affiliated Hospital of Guangxi Medical University; d Department of Bone and Joint Surgery, The First Affiliated Hospital of Guangxi Medical University, Nanning, China.

**Keywords:** colon adenocarcinoma, colorectal cancer, immune checkpoint inhibitors, immunotherapy, microRNA, tumor mutational burden

## Abstract

Some patients with advanced colon adenocarcinoma (COAD) are not sensitive to radiotherapy and chemotherapy, and as such, immunotherapy has become the most popular option for these patients. However, different patients respond differently to immunotherapy. Tumor mutational burden (TMB) has been used as a predictor of the response of advanced COAD patients to immunotherapy. A high TMB typically indicates that the patient's immune system will respond well to immunotherapy. In addition, while microRNAs (miRNA) have been shown to play an important role in treatment responses associated with the immune system, the relationship between miRNA expression levels and TMB has not been clarified in COAD.

We downloaded miRNA data and mutational files of COAD from the Cancer Genome Atlas database. Differentially expressed miRNAs were screened in the training group, and miRNAs used to construct the model were further identified using the LASSO logistic regression method. After building the miRNA-based model, we explored the correlation between the model and TMB. The model was verified by a receiver operating characteristic curve, and the correlation between it and 3 widely used immune checkpoints (programmed death receptor-1, programmed death-ligand 1, and cytotoxic T-lymphocyte associated protein-4) was explored. Functional enrichment analysis of the selected miRNAs was performed, and these respective miRNA target genes were predicted using online tools.

Our results showed that a total of 32 differentially expressed miRNAs were used in the construction of the model. The accuracies of the models of the 2 datasets (training and test sets) were 0.987 and 0.934, respectively. Correlation analysis showed that the correlation of the model with programmed death-ligand 1 and cytotoxic T-lymphocyte associated protein-4, as well as TMB, was high, but there was no correlation with programmed death receptor-1. The results of functional enrichment analysis indicated that these 32 miRNAs were involved in many immune-related biological processes and tumor-related pathways.

Therefore, this study demonstrated that differentially expressed miRNAs can be used to predict the TMB level, which can help identify advanced COAD patients who will respond well to immunotherapy. The miRNA-based model may be used as a tool to predict the TMB level in patients with advanced COAD.

## 1 Introduction

Colorectal cancer (CRC) is one of the most commonly diagnosed cancers worldwide, accounting for a third of the morbidity and mortality rates in both males and females. It is estimated that there will be 147,950 new CRC cases and 53,200 deaths from CRC in the United States in 2020.^[1]^ Colon adenocarcinoma (COAD), which is one type of CRC, represents the majority of all of the CRC cases in the United States.^[2]^ Traditional treatments for COAD mainly include surgery, radiotherapy, and chemotherapy. With early screening and effective treatment, a 5-year survival rate of 90% can be achieved.^[3]^ However, due to the subtle symptoms in the early stage, some patients have metastases at the time of initial diagnosis, resulting in a 5-year relative survival rate of only 14%, although they acquire systemic therapy.^[3]^ Therefore, it is urgent to explore effective treatment schemes for patients with advanced COAD.

Immunotherapy is to control the time and place of immune responses to increase antitumor activity through an immune checkpoint blockade. Immunotherapeutics, including high dose interleukin-2 and antibodies that block programmed death receptor-1 (PD-1)/programmed death-ligand 1 (PD-L1), and cytotoxic T-lymphocyte associated protein-4 (CTLA-4) can induce durable responses across numerous types of solid tumors^[4–10]^ and hematologic malignancies.^[11,12]^ Immunotherapy has been shown to be highly effective in cancer. The use of tumor PD-L1 expression as a biomarker has been studied extensively.^[13]^ However, there is a general need to better identify responders, as only 25% to 30% of patients under checkpoint treatment show long-term responses and these might not be exclusively identified by PD-L1 expression.^[14,15]^ It is an unmet need for biomarkers that will identify patients more likely to respond to PD-1/PD-L1 blockade as well as other immunotherapeutics.

Thus, identifying patients who are most likely to benefit from immunotherapy is crucial. Tumor mutational burden (TMB), measured by hybrid based NGS, is a new biomarker for response to immunotherapy.^[16,17]^ Cancers are caused by the accumulation of somatic mutations that can result in the expression of neoantigens.^[18]^ A high TMB can lead to modifications of the proteins encoded by the mutated genes, which would make them more easily recognized by the immune system because of the neoantigens.^[19]^ It has been suggested that tumors with numerous antigens are more sensitive to immune checkpoint inhibitors (ICIs)^[20,21]^ and have a higher TMB level, and have been shown to exhibit a strong response to immunity inhibitors in non-small cell lung carcinoma, melanoma, and CRC.^[18,22,23]^ High TMB can lead to modifications of the proteins encoded by the mutated genes. The modified proteins may be recognized by the immune system as “nonself” and activate specific.^[24]^ The translation of the mutated gene into a modified protein requires posttranscriptional regulation, and microRNAs (miRNAs) are important molecules involved in posttranscriptional regulation.

miRNA are a class of small RNAs with no coding potential. By complementary pairing to the 3′-untranslated region of messenger RNA, miRNAs exert posttranscriptional control of protein expression, which are often expressed aberrantly in cancer.^[25,26]^ Since miRNAs are involved in the regulation of various cancer hallmarks, miRNAs may be promising outcome predictors for various types of cancers.^[27–29]^ Studies have shown that miRNAs play important roles in mediating and controlling several immune and cancer cell interactions.^[30]^ Therefore, we hypothesized that miRNA expression patterns could be used as biomarker for predicting TMB levels different. To confirm our hypothesis, we downloaded the datasets of COAD, including mutation annotation files and miRNA expression profiles from the Cancer Genome Atlas (TCGA) database, in order to establish an miRNA-based model for predicting TMB levels in COAD.

## 2 Material and methods

### 2.1 Data acquisition and processing

The miRNA expression profiles contained 458 samples (450 COAD tissue samples and 8 matched healthy colon tissue samples) were obtained from the TCGA database (https://gdc.cancer.gov/). The mutation data of COAD were also downloaded from TCGA. All data were downloaded from public databases (TCGA), so ethical approval did not apply to this study. TMB was defined as the number of somatic variants per megabase (MB) of genome.^[31]^ This study estimated the size of the exome to be 38 MB.^[32]^ We chose the Varscan2 pipeline as the somatic mutation calling workflow. We also defined those genes with mutations <10 per MB as a low TMB level and with ≥10 mutations per MB as a high TMB level.^[33]^ In total, 383 samples (309 low TMB samples and 74 high TMB samples) with miRNA profiles and TMB expression values were identified for further analysis using the “limma” package in R (version 4.0.0; https://www.r-project.org/). These samples were divided into the training set (60%) and test set (40%) using “caret” package based on clinical characteristics (Table 1, all *P*-value > .05) after using bootstrapping method.

**Table 1:** Information of the training and test sets.

	Test set		Training set		
Characteristic	Numbers	%	Numbers	%	*P*
Age					
≤ 65 yr	64	41.83%	99	43.04%	.896
> 65 yr	89	58.17%	131	56.96%	
Gender					
Female	77	50.33%	111	48.26%	.770
Male	76	49.67%	119	51.74%	
Stage					
Stage I-II	86	56.21%	122	53.04%	.641
Stage III-IV	63	41.18%	101	43.91%	
Unknown	4	2.61%	7	3.04%	
T					
T1–2	28	18.3%	43	18.7%	1
T3–4	125	81.7%	186	80.87%	
Unknown	0	0%	1	0.43%	
M					
M0	108	70.59%	160	69.57%	.531
M1	27	17.65%	32	13.91%	
Unknown	18	11.76%	38	16.52%	
N					
N0	92	60.13%	129	56.09%	.497
N1–3	61	39.87%	101	43.91%	

### 2.2 Screening of differentially expressed miRNAs

The miRNAs that were expressed <10% in the COAD samples were excluded from the training set. The differentially expressed miRNAs in the training set were analyzed using the “limma” package.^[34]^ The fold change (FC) in the expression of each miRNA was calculated, and the miRNAs that met the requirement of |logFC| > |log1.5| (FC = 1.5) and *P*-value < .01 (adjusted by false discovery rate) were considered as differentially expressed. The expression of miRNA was visualized in a heatmap using “pheatmap” package.

### 2.3 miRNA-based model for predicting the TMB level

Least absolute shrinkage and selection operator (LASSO) regression, which was used to reduce the dimension in multiple highly correlated features, helped with the selection of optimal miRNAs in this study. Based on the remaining miRNAs weighted by their own coefficients in LASSO regression, we constructed a model to estimate TMB levels. A model index for each sample could be created by the following formula:

Index = Intercept + Exp1 ∗ Coef1 + Exp2 ∗ Coef2 + Exp3 ∗ Coef3 + …

where the “Intercept” is a constant of the created model, “Exp” represents the expression value of a selected miRNA, and “Coef” represents the respective weighting coefficient. An index of ≥0.5 was considered as a high TMB level, and an index of <0.5 was considered to be low TMB. The above steps were completed with the “glmnet” package.^[35]^ We performed the validation in the test set to estimate the accuracy and applicability of the prediction model. The efficiency of the model was assessed by 5 frequently used aspects of accuracy: sensitivity, specificity, positive predictive value, negative predictive value, and area under curve (AUC). ROC curves were drawn and compared using the “pROC” package^[36]^ in R.

### 2.4 Principal component analysis (PCA)

In order to make the model as recapitulative and low-dimensional as possible, we performed PCA within gene profiles of differentially expressed miRNAs before and after the feature dimension reduction in LASSO. The above steps were performed using the “ggplot2” package in R. The outputs of the PCA are shown in 2-dimensional scatter plots (Fig. 3B and C).

### 2.5 Correlation between the miRNA-based model and the expression of 3 immune checkpoints and TMB levels

In the total set, the model index of each sample was calculated. We then estimated the linear relationship between the model and TMB, as well as the expression of 3 widely known immune checkpoints (PD-1, PD-L1, and CTLA-4) using the “limma,” “ggplot2,” and “ggpubr” packages in R.

### 2.6 Gene ontology (GO) and Kyoto Encyclopedia of Genes and Genomes (KEGG) enrichment analysis

DIANA-mirPath web-server45 (http://snf-515788.vm.okeanos.grnet.gr/) was used to perform KEGG pathway and GO enrichment analysis for selected miRNAs of the model. The TarBase 7.0^[37]^ tool in the DIANA-mirPath web-server was utilized in this study. A *P*-value of <0.01 was considered to be significantly enriched. The results of GO and KEGG pathway analysis were visualized in the bubble plots using the“ggplot2” package in R.

### 2.7 Target genes of selected miRNA

The MiRDB (http://mirdb.org/), miRTarBase (http://mirtarbase.mbc.nctu.edu.tw/php/index.php), and TargetScan (http://www.targetscan.org/vert_72/) databases were applied to these miRNAs in order to investigate their target genes. We identified these genes that were simultaneously recognized by the 3 above database as target genes of selected miRNAs. The results were also visualized in Venn diagrams using the “VennDiagram” package in R.

## 3 Results

### 3.1 Differentially expressed miRNAs

**Figure 1:**
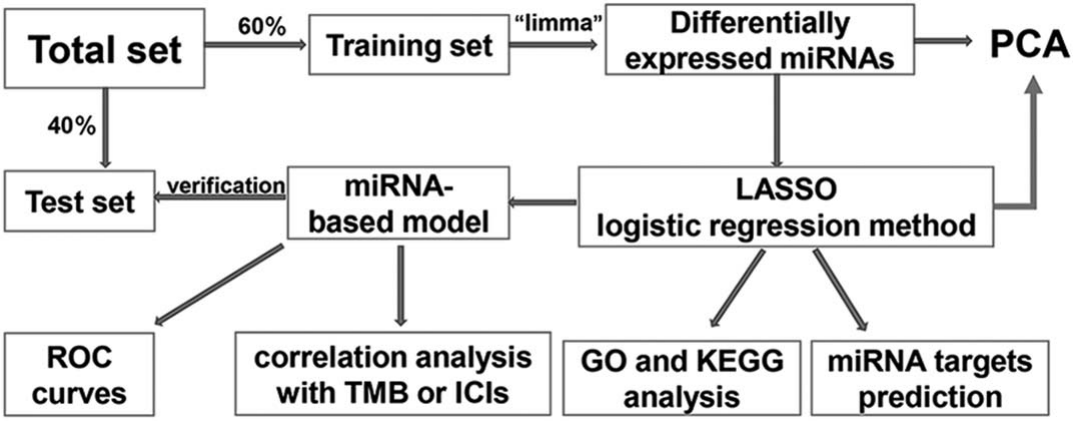
Workflow of the present study.

**Figure 2:**
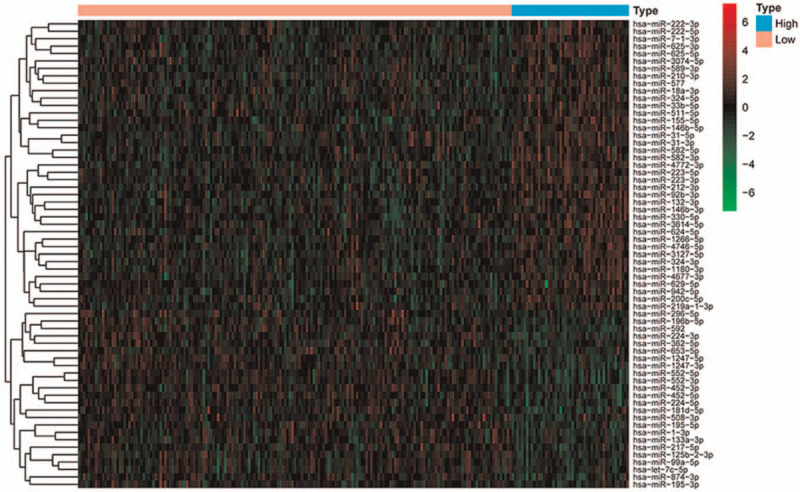
A heatmap of differently expressed microRNAs that can distinguish between a high TMB and a low TMB in patients with COAD. COAD = colon adenocarcinoma, TMB = tumor mutational burden.

The workflow of our research is shown in Figure 1. Conventional clinicopathological characteristics did not differ significantly between the training sets and the test sets (Table 1). There were 230 samples in the training sets, including 181 with low TMB levels and 49 with high TMB levels. A total of 63 differentially expressed miRNAs, including 39 upregulated miRNAs and 24 downregulated miRNAs, met the cut-off criteria (*P* < .01 and |logFC| > |log1.5|) in the training set. Figure 2 shows a heat map representing the results of the differentially expressed analysis. The expression values of differently expressed miRNAs were related to the TMB level of the samples, which could distinguish the samples with high expression of TMB from those with low expression.

### 3.2 PCA and feature selection

**Figure 3:**
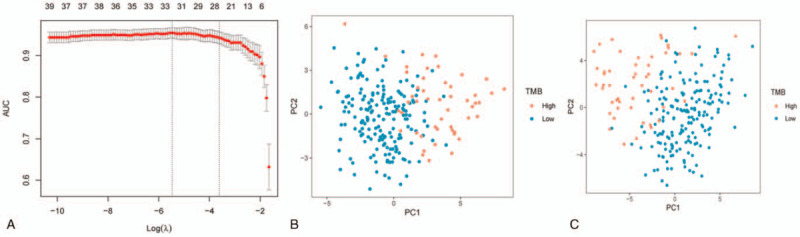
LASSO regression model and principal component analysis. (A) A 10-fold cross-validation for tuning parameter selection in the LASSO model. (B) PCA before and (C) after LASSO variable reduction. LASSO = least absolute shrinkage and selection operator, PCA = principal component analysis.

In order to develop a miRNA-based model to predict the TMB level in CRC patients, this study performed a LASSO logistic regression method using the expression data of the 63 miRNAs in the training sets. We assessed the identification and classification accuracies via 10-fold cross-validations. A total of 32 miRNAs with nonzero regression coefficients were identified as the most ideal characteristics (Fig. 3A). These 32 miRNAs included miR-223-5p, miR-874-3p, miR-222-3p, miR-625-3p, miR-330-5p, miR-196b-5p, miR-99a-5p, miR-1-3p, miR-552-5p, miR-653-5p, miR-3614-5p, miR-624-5p, miR-1266-5p, miR-155-5p, miR-296-5p, miR-222-5p, miR-4772-3p, miR-146b-5p, miR-582-3p, miR-217-5p, miR-629-5p, miR-1247-5p, miR-1247-3p, miR-452-5p, miR-4746-5p, miR-195-3p, miR-589-3p, miR-92b-3p, miR-452-3p, miR-7-1-3p, miR-362-5p, and miR-212-3p. The PCA results based on all of the 63 differently expressed miRNAs are shown in Figure 3B. The PCA results of the 32 miRNAs identified based on the LASSO method are shown in Figure 3C. Figure 3C shows that the 32 remaining miRNAs exhibited a better discriminatory ability across samples with different TMB levels.

### 3.3 Construction of the miRNA-based model

**Figure 4:**
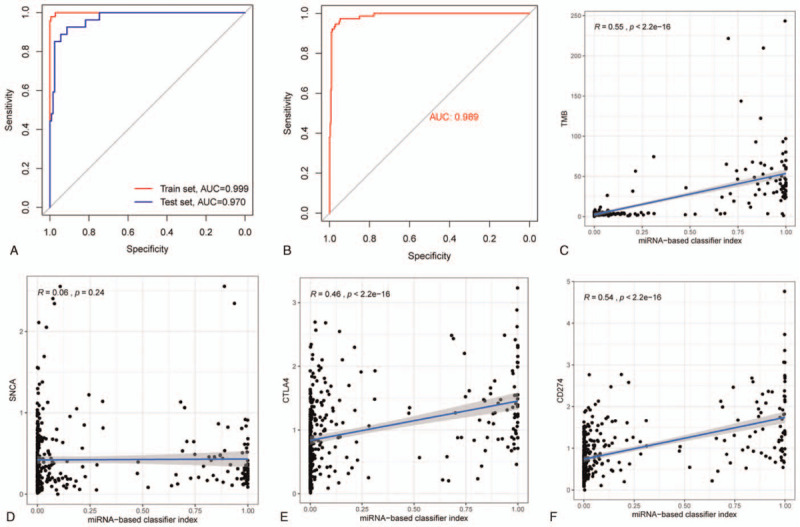
ROC curves for the 32-miRNA-based model and its correlation with TMB, PD-1, PD-L1, and CTLA-4. (A) ROC analyses in the training and test sets. (B) ROC in the total set. (C) The 32-miRNA-based model correlated highly with TMB. (D) The 32-miRNA-based model shows no correlation with PD-1 expression. (E) The 32-miRNA-based model showed high correlation with PD-L1 expression. (F) The 32-miRNA-based model was highly correlated with CTLA-4 expression. CTLA-4 = cytotoxic T lymphocyte-associated antigen-4, PD-1 = programmed cell death-1, PD-L1 = programmed cell death-Ligand 1, ROC = receiver operating characteristic.

The results of the PCA of the 32 miRNAs identified based on the LASSO method are shown in Figure 3C. Based on the above formula, the study constructed an miRNA-based model where the intercept equaled –20.6387343620101. A test set validated the accuracy of the model. The accuracy of the 32 miRNA-based model was 0.966 in the total set, and 0.987 and 0.924 in the training set and test set, respectively (Fig. 4A and B, Table 2). The accuracy, including the sensitivity, specificity, positive predictive value, negative predictive value, and AUC values verified that the model had a high sample recognition efficiency (Table 2). The AUC of the receiver operating characteristic (ROC) curve analysis of the training set and the test set were 0.999 and 0.970, respectively. There were no significant differences between the test and training sets (Fig. 4A).

**Table 2:** Performance of 32-miRNA-based model in colon adenocarcinoma.

Cohort	Se	Sp	PPV	NPV	Accuracy	AUC
Train	0.959	0.994	0.979	0.989	0.987	0.999
Test	0.851	0.952	0.793	0.967	0.934	0.970
Total	0.921	0.977	0.909	0.980	0.966	0.988

### 3.4 Connection between the miRNA-based model and 3 ICIs and TMB levels

As expected, the 32 miRNA-based model displayed a strong association with TMB (*R* = 0.55, *P* < 2.2e−16, Fig. 4C). The results also showed that the model had a high association with PD-L1 and CTLA-4 (*R* = 0.54, *P* < 2.2e−16; *R* = 0.46, *P* < 2.2e−16; respectively; Fig. 4E and F). However, the model was not associated with PD-1 (*R* = 0.06, *P* = .24, Fig. 4D).

### 3.5 Enrichment analysis

**Figure 5:**
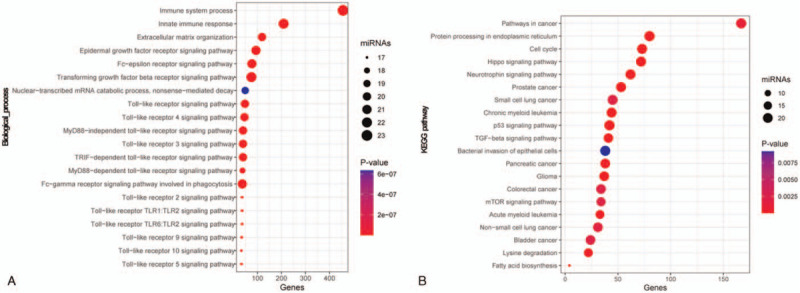
GO and KEGG enrichment analysis of the 32 miRNAs. (A) Significantly enriched immune-related biological process and (B) significantly enriched cancer-related pathways. GO = gene ontology, KEGG = Kyoto Encyclopedia of Genes and Genomes.

The GO and KEGG enrichment analysis of these 32 miRNAs are shown in Figure 5A and B, from which it could be seen that they were enriched in numerous immune-related biological processes, as well as cancer-related pathways. This result suggested that these miRNAs play nonnegligible roles in cancer-related immune processes.

### 3.6 Prediction of the target genes of the 32 miRNAs via miRDB, miRTarBase, and TargetScan databases

**Figure 6:**
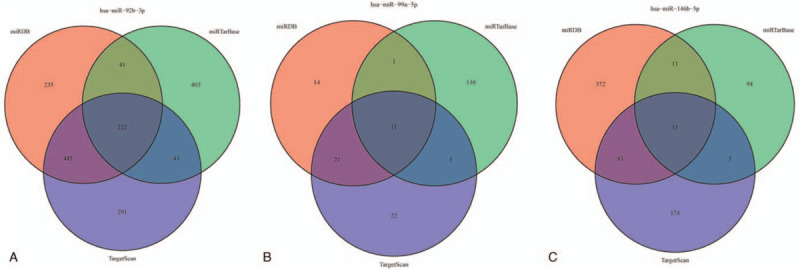
The Venn diagrams of differently expressed microRNAs based on miRDB, miRTarBase, and TargetScan.

After a total of 32 miRNA were identified by the LASSO regression method, we visualized the forecast results of the above 3 databases via drawing Venn diagrams. Certain genes were identified as the target genes of those selected miRNAs, including miR-92b-3p, miR-99a-5p, and miR-146b-5p (Fig. 6A–C).

## 4 Discussion

Although immunotherapy shows a satisfactory curative effect for tumor therapy, only a fraction of patients benefit from it. One reason for the low success rate may be that low TMB levels cannot trigger a proper immune response in the body. A previous study showed that high somatic mutation loads were associated with prolonged progression-free survival.^[38]^ Further research found that the large proportion of mutant neoantigens in mismatch repair deficient cancers make them sensitive to immune checkpoint blockade, regardless of the tissue origin of the cancer cells.^[39]^ The TMB, as a genomic marker and predictor of ICIs treatment response, has been reported in many cancers, such as lung cancer^[40]^ and bladder cancer.^[41]^ Thus, predicting the TMB levels of patients with cancer will allow for a more personalized treatment plan.

Previous studies have found that miRNAs simulate the therapeutic efficacy of ICIs via regulating the expression of checkpoint receptors either directly or indirectly.^[25]^ Recent advances have revealed that miRNAs are being recognized as an important role in ICI therapy. Some studies have begun to highlight the prognostic value of a miRNA-based model for CRC.^[42]^ However, it is unknown if miRNA expression is related to TMB in COAD. Therefore, we hypothesized that miRNA expression could be used as a biomarker to predict the TMB level in advanced COAD patients, which would allow clinicians to identify patients who are more sensitive to immunotherapy. Thus, we established a miRNA-related model to verify this hypothesis. We identified 32 miRNAs for model construction. The ROC curves showed that this model was very accurate for predicting the TMB level in both the training and test sets (Fig. 4). For this result, we believe that the accuracy of this 32 miRNA-based model was so high because of the typical COAD cases in TCGA and the relatively large number of miRNAs screened. Therefore, external verification that contains more cases are necessary in the future. The PCA showed that the 32 selected miRNAs were able to distinguish patients with different TMB levels in 2 dimensions, which was similar to the results of the PCA of the 63 differentially expressed miRNAs. This indicated that the miRNA-based model was robust and available.

The results of the correlation analysis between the model index and the 3 immune checkpoints showed that this model has a positive correlation with PD-L1 and CTLA-4, but had no correlation with PD-1. This discovery aroused our interest. PD-L1 expression is strictly associated with miRNAs function in cancer cells.^[43]^ Cancer cells highly express PD-L1 which help them in evading immune responses.^[44]^ PD-L1 is a transmembrane protein, highly expressed on antigen presenting cells and is involved in imparting self tolerance. A previous study showed that certain miRNA expression could decrease PD-L1 expression in patients with COAD.^[45]^ In addition, Several studies also have revealed that miRNAs could decrease PD-L1 expression by binding to 3′-untranslated region of PD-L1, suggesting that miRNAs were negatively related with PD-L1 expression.^[46–48]^ Moreover, miRNAs regulate PD-L1 expression and have potential therapeutic uses. For instance, miR-200/ZEB axis is strongly correlated with high PD-L1 expression, consistent annihilation of CD8+ cell infiltration, and high EMT scores.^[49]^ Another miRNA, miR-197-5p is negatively correlated with PD-L1 expression via CDC28 protein kinase regulatory subunit 1B/STAT3 axis.^[50]^ Study had shown that circFGFR1 could directly interact with miR-381-3p and subsequently act as a miRNA sponge to upregulate the expression of the miR-381-3p target gene C-X-C motif chemokine receptor 4, which promoted non-small cell lung carcinoma progression and resistance to anti-programmed cell death 1-based therapy.^[51]^ However, in our research, this miRNA expression signature was positively related with PD-L1 expression. Besides, there are few related literatures between miRNAs and PD-1, and the regulatory network between them is still unclear. Study have found that the miRNAs were positively related with PD-1 expression in CRC,^[52]^ But it was reversed in another study.^[53]^ This reveals that the relationship between miRNAs and PD-1 expression is still controversial. And the result of our research may be another relationship between miRNAs and PD-1. Thus, future researches are required to explore the underlying mechanism between these TMB-related miRNAs and PD-L1 and PD -1 expression. Besides, the miRNA-related expression signature showed a median positive correlation with TMB, indicating that this miRNA-related expression signature predicted the TMB level from a biological perspective of the anticancer immune response. This result was consistent with our expectations.

GO analysis demonstrated that 32 miRNAs were involved in a number of important biological processes associated with the immune response, such as “immune system process,” “innate immune response,” and “toll-like receptor signaling pathway.” KEGG analysis indicated that the 32 miRNAs were also involved in a relatively unique pathway associated with various human tumors, such as “ CRC,” bladder cancer,” and “pancreatic cancer.” These initial results indicated that the miRNA-based model was feasible for predicting TMB levels in patients with COAD. Although this result is exciting, more research is needed to verify this result.

Although this miRNA-based model showed excellent results, there are still several potential limitations in the present research. First, the threshold of TMB levels may vary owing to different methods.^[54]^ Second, the number of miRNAs in this model is large compared with another study,^[33]^ which may be responsible for the high accuracy of ROC curves. Third, further studies with a larger sample size are needed in order to validate the forecast effect of the signature of the 32 miRNAs. Fourth, further validation of the selected miRNA target genes is required.

## 5 Conclusions

We demonstrated that the differential expression patterns of 32 miRNAs have a high correlation with the TMB values of COAD patients. The miRNAs-based model may provide clinicians with a predictor of TMB levels in advanced COAD patients. The results from this study have the potential to help distinguish patients with a high TMB who will benefit from immunotherapy.

## Acknowledgments

We thank the authors who provided the data for this study. We thank LetPub (www.letpub.com) for its linguistic assistance during the preparation of this manuscript.

## Author contributions

**Data curation:** Zhengtian Li, Lingling Jiang, Jun Huang, Bo Zhang.

**Formal analysis:** Zhengtian Li, Rong Zhao, Jun Huang, Wenkang Yang, Zhenpei Wen, Bo Zhang, Gang Du.

**Funding acquisition:** Gang Du.

**Software:** Zhengtian Li, Jun Huang, Zhenpei Wen.

**Validation:** Zhengtian Li, Wenkang Yang.

**Visualization:** Zhengtian Li, Lingling Jiang.

**Writing – original draft:** Zhengtian Li, Lingling Jiang, Rong Zhao.

**Writing – review & editing:** Zhengtian Li.
